# Gastrointestinal Stromal Tumor Induced Hypercalcemia

**DOI:** 10.1155/2017/4972017

**Published:** 2017-04-06

**Authors:** Aram Barbaryan, Stefania Bailuc, Padma Poddutoori, Aida Richardson, Aibek E. Mirrakhimov

**Affiliations:** ^1^Department of Internal Medicine, University of Kansas Medical Center, Kansas City, KS, USA; ^2^Department of Medicine, HSHS Saint Mary's Hospital, Decatur, IL, USA; ^3^Department of Pathology and Laboratory Medicine, University of Kansas Medical Center, Kansas City, KS, USA; ^4^Department of Medicine, University of Kentucky, Lexington, KY, USA

## Abstract

Hypercalcemia in patients with cancer is a common laboratory finding affecting up to 44% of that patient population. 1,25-Dihydroxyvitamin D_3_ mediated hypercalcemia is one of the rare mechanisms of this endocrine emergency in cancer patients. It is even rarer for solid organ neoplasms to present with hypercalcemia mediated through the production of 1,25-dihydroxyvitamin D_3_. We report a case of a 77-year-old female who presented to the hospital with hypercalcemia and later was found to have metastatic gastrointestinal stromal tumor. There have been only 5 cases of gastrointestinal stromal tumor described in literature resulting in hypercalcemia. In our case, the mechanism of hypercalcemia was thought to be related to overproduction of 1,25-dihydroxyvitamin by tumor cells. The patient had a favorable response to imatinib with normalization of serum calcium level. Unfortunately, she developed fluid retention due to imatinib which was discontinued resulting in relapse of hypercalcemia that was resistant to all other treatment options.

## 1. Introduction

Hypercalcemia of malignancy is the most common cause of hypercalcemia in hospitalized patients, while primary hyperparathyroidism is the most common cause in ambulatory patients. Hypercalcemia in patients with cancer is a common laboratory finding affecting up to 44% of that patient population [1–3]. It is common in advanced stages of cancer pertaining poor prognosis and survival [4]. The most common (80%) mechanism is through secretion of parathyroid hormone related peptide (PTH-rP) by tumors cells followed by bone metastasis in the remaining majority of cases (20%). Much less common mechanisms (<1% combined) are related to secretion of 1,25-dihydroxyvitamin D_3_ (1,25(OH)_2_D_3_ or calcitriol) and parathyroid hormone (PTH) by tumors cells [5].

## 2. Case Presentation

A 77-year-old female with history of hypertension presented to the hospital with the chief complaint of progressive weakness and confusion which started 3-4 days prior to presentation. Patient denied any other complaints. Her vital signs were within normal limits. Her physical examination was unremarkable. Her only home medication was lisinopril for hypertension. Upon initial investigation, she was found to have hypercalcemia of 15 mg/dL (reference range: 8.5–10.6 mg/dL) and acute kidney injury (AKI) with creatinine of 1.76 mg/dL (reference range: 0.4–1.0). The rest of the comprehensive metabolic profile was normal.

Patient was started on intravenous (IV) fluids for the management of hypercalcemia and AKI and the work-up of hypercalcemia was initiated. Parathyroid hormone (PTH), PTH-rP, 25(OH)D_3_, calcitriol, and urine and serum electrophoresis were ordered. PTH was 7.9 pg/mL (reference range: 10–65), PTH-rP was normal, 25(OH)D_3_ was normal, and calcitriol was elevated at 129 (reference range 19.9–79.3 pg/mL). Serum and urine electrophoresis were normal.

Patient underwent computer tomography (CT) of the chest, abdomen, and pelvis to further investigate the cause of hypercalcemia. CT chest was normal, but noncontrast CT of abdomen showed multiple hepatic lesions and diffuse nodular thickening of the peritoneum consistent with peritoneal carcinomatosis. PET/CT scan of the neck, chest, abdomen, and pelvis demonstrated widespread hepatic and peritoneal metastatic disease with small bilateral hypermetabolic pleural effusions. Due to excessive metastasis the primary location of tumor was not identified.

**Figure 1:**
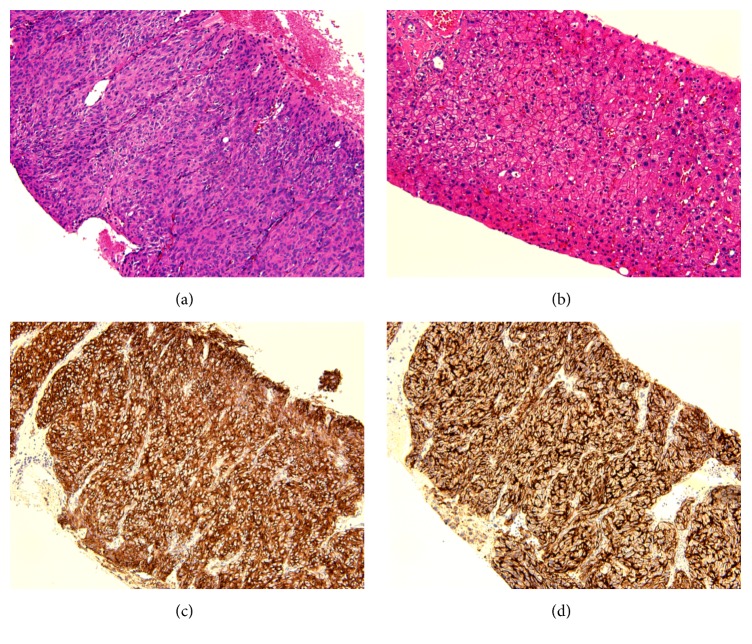
20x micrographs, (a–d). Spindle cell neoplasm (a) is more cellular when compared to surrounding liver tissue (b). The tumor cells of spindle cell neoplasm (a) appear uniform, with monomorphic nuclei and eosinophilic cytoplasm. Tumor cells stain strongly positive for CD117 (c) and DOG1 (d).

Patient underwent ultrasound guided percutaneous biopsy of liver showing spindle cell neoplasm, consistent with gastrointestinal stromal tumor (GIST). Immunohistochemical stains showed that tumor cells are positive for CD117 and DOG1 and negative for pancytokeratin, hepatocytic specific antigen, S-100, and SMA, supporting the above diagnosis (Figure 1). The specimen was also evaluated for GIST gene mutation analysis showing the presence of KIT gene mutation (affecting exon 9 c.1502_1503insTGCCTA/p.Tyr503_Phe504insAlaTyr) and absence of PDGFRA mutation.

Initially hypercalcemia was treated with IV fluids; after diagnosis of metastatic GIST was made, imatinib (400 mg once daily and then advanced to 400 mg twice daily) was started. Upon discharge, patient's Ca level and vitamin D and kidney function were normalized. Repeat PET/CT showed interval decrease in FDG uptake of hepatic metastatic lesions. Since the initial hospitalization, she has had multiple readmissions for fluid retention related to imatinib. Patient was taken off imatinib because of side effects resulting in relapse of hypercalcemia and increase in calcitriol that did not respond to trial of calcitonin, steroids, zoledronic acid, and denosumab. In the meantime, patient was started on nilotinib (second-generation Tyrosine Kinase Inhibitor) but unfortunately her condition continued to deteriorate and she was subsequently transitioned to hospice care.

## 3. Discussion

Vitamin D3 (cholecalciferol) is synthesized in the skin under the influence of ultraviolet light from 7-dehydrocholesterol [6]. Subsequently, Vitamin D3 bound to vitamin D binding protein enters the circulation and undergoes hydroxylation to 25-hydroxyvitamin D3 (25(OH)D_3_) in the liver through the action of vitamin D_3_-25-hydroxylase enzyme [7]. 25(OH)D_3_ is the major circulating form of vitamin D3. In the kidneys enzyme 25-hydroxyvitamin D_3_-1-hydroxylase (PTH dependent action) converts 25(OH)D_3_ to its metabolically active form 1,25-dihydroxyvitamin D_3_ (calcitriol) [8]. Calcitriol increases calcium and phosphorus absorption from intestine (duodenum and proximal small intestine) and also promotes mobilization of calcium from bone [9, 10].

Excessive production of vitamin D metabolites is one of the mechanisms of vitamin D induced hypercalcemia. Ectopic (extrarenal) production of calcitriol is the main mechanisms of hypercalcemia in granulomatous diseases (like sarcoidosis and tuberculosis) and lymphomas. In these cases, 25-hydroxyvitamin D_3_-1-hydroxylase present in pulmonary alveolar macrophages and lymphocytes is responsible for conversion of 25(OH)D_3_ to 1,25(OH)_2_D_3_ [11–15]. Sarcoidosis was the most common cause of calcitriol mediated hypercalcemia in the largest cohort of patients. Hematological malignancies (17%) and infectious (8%) and solid organ malignancies (5%) represented the less common etiologies [16].

Our search identified only 5 cases of GIST related hypercalcemia in the English literature. In two of those cases the mechanism of hypercalcemia was thought to be related to overproduction of calcitriol, in one case the production of PTH-rP was the cause, and in the remaining two cases no mechanism was identified. [17–21]. In one of those cases GIST gene mutation analysis showed the presence of KIT gene mutation affecting exon 9 (no data was available regarding the type of mutation) and absence of PDGFRA mutation [19]. No molecular profile data were found in the remaining four cases [17, 18, 20, 21]. In our case the two most common causes of hypercalcemia of malignancy such as secretion of PTH-rP and bone metastasis were excluded by negative PTH-rP values and PET-CT scan. Hyperparathyroidism was also ruled out by decreased level of PTH. Other cancers and multiple myeloma were ruled out by negative results of PET-CT scan and the absence of monoclonal protein in serum and urine. The only mechanism that could explain hypercalcemia in our case was increased production of calcitriol by tumor cells. The proof of this theory is elevated level of calcitriol upon initial presentation that was corrected posttreatment with imatinib followed by relapse of hypercalcemia and increased serum concentration of calcitriol when patient was taken off imatinib due to side effects.

GISTs are mesenchymal neoplasms that most commonly arise in the stomach and small intestine and comprise only 1% of cancers primarily arising in the gastrointestinal tract [22, 23]. The annual incidence in the United States is 4000–6000 cases, with average age of diagnosis being 63 [24]. As a group (>90%) they are defined by activating mutations in KIT gene producing significant amounts of KIT transmembrane receptor tyrosine kinase (RTK) [25]. However, in minority of cases GISTs are KIT negative; in those cases activating mutations in another gene, the platelet-derived growth factor receptor alpha (PDGFRA) genes are responsible for encoding an RTK [23]. Most commonly they arise from the stomach (40–60%) and jejunum/ileum (25–30%). Duodenum, esophagus, and colorectum are less common sites [24]. Before 2001, surgery was the only available treatment for patients with GIST, but management of GIST dramatically improved since the understanding of the role of KIT/PDGFRA genes in the molecular pathogenesis of GISTs leading to effective systemic therapy with small molecule inhibitors of tyrosine kinase receptors of which imatinib is considered prototype drug [26–28].

## 4. Conclusion

Hypercalcemia from gastrointestinal stromal tumor is a rare but serious endocrine emergency which needs immediate treatment. Tyrosine Kinase Inhibitors are the recommended initial treatment for metastatic gastrointestinal stromal tumor with KIT gene mutation. Our patient had a decrease in size of metastatic lesions and calcitriol levels after therapy with imatinib. Hence early diagnosis and GIST gene mutation analysis are crucial in initiating appropriate treatment.
